# Adhesion of Platelets to Colon Cancer Cells Is Necessary to Promote Tumor Development in Xenograft, Genetic and Inflammation Models

**DOI:** 10.3390/cancers13164243

**Published:** 2021-08-23

**Authors:** Marica Cariello, Elena Piccinin, Roberta Zerlotin, Marilidia Piglionica, Claudia Peres, Chiara Divella, Anna Signorile, Gaetano Villani, Giuseppe Ingravallo, Carlo Sabbà, Antonio Moschetta

**Affiliations:** 1 Department of Interdisciplinary Medicine, “Aldo Moro” University of Bari, 70124 Bari, Italy; marica.cariello@uniba.it (M.C.); elena.piccinin@uniba.it (E.P.); roberta_zerlotin@libero.it (R.Z.); marilidia.piglionica@uniba.it (M.P.); carlo.sabba@uniba.it (C.S.); 2 Department of Basic Medical Sciences, Neurosciences and Sense Organs, “Aldo Moro” University of Bari, 70124 Bari, Italy; anna.signorile@uniba.it (A.S.); gaetano.villani@uniba.it (G.V.); 3 INBB, National Institute for Biostructures and Biosystems, 00136 Rome, Italy; claudiaperes18@gmail.com; 4 Nephrology, Dialysis, and Transplantation Unit, Department of Emergency and Organ Transplantation, University of Bari Aldo Moro, 71024 Bari, Italy; claretta.divella@libero.it; 5 Pathology Section, Department of Emergency and Organ Transplantation, University of Bari Aldo Moro, 70124 Bari, Italy; giuseppe.ingravallo@uniba.it

**Keywords:** colon cancer, platelets, P-selectin

## Abstract

**Simple Summary:**

Platelets are small, anucleate, metabolically active cells and they represent an important linkage between tissue damage and inflammatory response. Several studies focused on the central role of platelets in inflammation and tumor development through their direct interaction with other cell types. Mice lacking the vascular adhesion molecules P-selectin exhibited a reduction in tumor metastases. We demonstrated that P-selectin-ablated platelets reduced tumor growth in a xenograft adenocarcinoma model. Furthermore, the lack of P-selectin decreased colon cancer progression in genetic mouse models and in chemically-induced colitis colorectal carcinogenesis. Our results suggest that platelets-cancer cells crosstalk via P-selectin is fundamental for tumor development.

**Abstract:**

Platelets represent the linkage between tissue damage and inflammatory response with a putative role in tumorigenesis. Given the importance of the microenvironment in colon cancer development, we elucidated the eventual role of platelets-cancer cells crosstalk in in vivo colon cancer models. To evaluate the involvement of platelets in intestinal tumorigenesis, we first analyzed if the ablation of β-integrin P-selectin that drives platelets-cell adhesion, would contribute to platelets-colon cancer cell interaction and drive cancer progression. In a xenograft tumor model, we observed that when tumors are inoculated with platelets, the ablation of P-selectin significantly reduced tumor growth compared to control platelets. Furthermore, in genetic models, as well as in chronic colitis-associated colorectal carcinogenesis, P-selectin ablated mice displayed a significant reduction in tumor number and size compared to control mice. Taken together, our data highlights the importance of platelets in the tumor microenvironment for intestinal tumorigenesis. These results support the hypothesis that a strategy aimed to inhibit platelets adhesion to tumor cells are able to block tumor growth and could represent a novel therapeutic approach to colon cancer treatment.

## 1. Introduction

The crosstalk between cancer cells and the microenvironment has a pivotal role in tumor development. Several studies focused on the involvement of platelets in cancer development and metastatization [1]. Platelets are produced from megakaryocytes in the bone marrow and are key regulators of hemostasis, repair and inflammation [2]. Platelets are anucleate, metabolically active cells provided with endoplasmic reticulum, Golgi apparatus, mitochondria and granules [3], and they are able to translate mRNAs into proteins [4,5]. Platelets can affect tumor growth and metastasis via several mechanisms, including the release of growth factors and cytokines, such as TGF-β, PDGF [6] and pro-inflammatory lipid signaling via PAF [7], the enhancement of the adhesion of cancer cells to the endothelium and the direct interaction with cancer cells. Specifically, platelet aggregates can prevent tumor cell death, creating a physical shield around tumor cells that protects them from natural killer cell-mediated death facilitating tumor cell dissemination into the bloodstream [8,9]. Moreover, Labelle et al. demonstrated that the platelets-colon cancer cells crosstalk and the production of TGF-β promote metastasis formation in the lung by inducing the epithelial-mesenchymal like transition (EMT) [10]. At the same time, cancer cells can assimilate platelet-derived proteins (platelets mimicry), enhancing the hematogenous dissemination of tumors [11].

P-selectin (CD62P) belongs to the selectin family and it is a cell adhesion molecule stored on platelets α-granules and Weibel-Palade bodies [12]. After inflammatory or thrombogenic stimuli, P-selectin translocates on the membrane surface mediating transient platelets contact with vascular endothelial cells [13]. Alves et al. observed that CD44 on LS174T colon cancer cells is the functional ligand of platelets P-selectin [14]. Several studies have shown that P-selectin deficient mice displayed a lower number of metastatic cells compared to wild-type mice [15,16]. Mice injected with colon cancer cells without P-selectin ligands presented a strong reduction of tumor metastases. Moreover, in human cancer cell lines, it has been demonstrated that P-selectin mediated the platelets’ adhesion to cancer cells [17], highlighting the key role of P-selectin in platelets cancer-cells crosstalk.

In the present work, we elucidated the eventual role of platelets-cancer cells crosstalk in in vivo models of colon cancer. In order to evaluate the involvement of platelets in intestinal tumorigenesis, we analyzed if the ablation of P-selectin would contribute to platelets-colon cancer cell interaction and reduce colon cancer progression.

## 2. Results

### 2.1. Platelets-Intestinal Cells Crosstalk in Human Intestine

**Figure 1:**
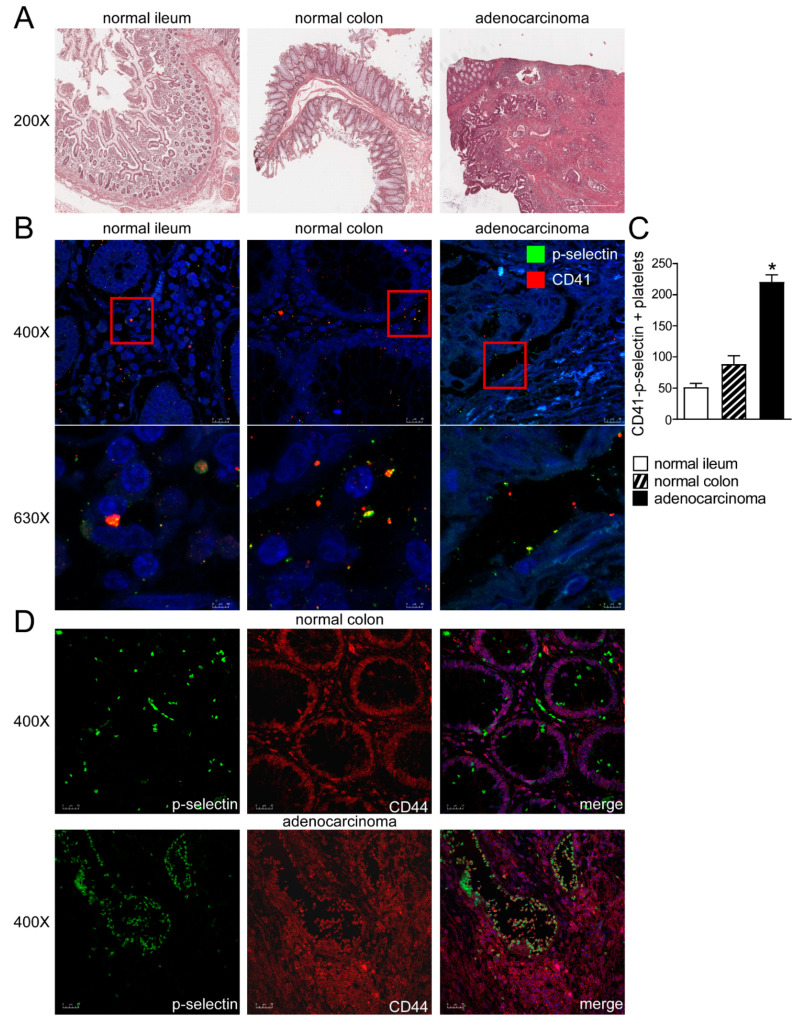
Platelets-intestinal cells crosstalk in human intestine. (**A**) Histology was assessed by H&E staining and was observed by light microscopy (magnification, 200×) in normal ileum, colon and colon adenocarcinoma. Representative specimens are shown. (**B**) The protein expression of P-selectin and CD41 was investigated by immunofluorescence and confocal microscopy analysis in paraffin-embedded sections from normal portions of ileum and colon and colon adenocarcinoma. P-selectin in green and CD41 in red. Representative images are shown. Magnification 630×. (**C**) CD-41/P-selectin positive platelets were quantified by ImageJ software. Results are expressed as mean ± SEM (* *p* < 0.05). (**D**) The protein expression of P-selectin and CD44 was investigated by immunofluorescence and confocal microscopy analysis in paraffin-embedded sections from normal portions of colon and colon adenocarcinoma. P-selectin in green and CD44 in red. Representative images are shown. Magnification 400×.

To evaluate the platelets-colon cancer cells crosstalk, we analyzed human colon adenocarcinoma, normal ileum and normal colon by H&E (Figure 1A). We assayed by P-selectin and CD41 (platelets marker) immunofluorescence human colon adenocarcinoma together with ileum and colon from control subjects. In normal ileum and colon, we observed the presence of platelets in close proximity to intestinal cells, whereas in adenocarcinoma platelets, aggregates along the intestinal epithelium, thus indicating the strong presence of platelets in the tumor microenvironment and the platelets-intestinal cells crosstalk in cancer (Figure 1B,C). In order to observe platelets-cancer cells crosstalk we performed CD44-P-selectin immunofluorescence in human healthy colon and colon adenocarcinoma. In normal colon we observed the expression of CD44 in colon cells and the presence of platelets (marked with P-selectin) in close proximity to cells. In adenocarcinoma, we detected platelets aggregates along the intestinal epithelium and a co-localization of CD44/P-selectin, thus indicating the strong presence of platelets in the tumor microenvironment and the platelets-intestinal cells crosstalk in cancer (Figure 1D, Supplementary Figure S1).

### 2.2. Platelets Isolated from P-selectin KO Mice Reduce Tumor Growth in Xenograft Adenocarcinoma Model

**Figure 2:**
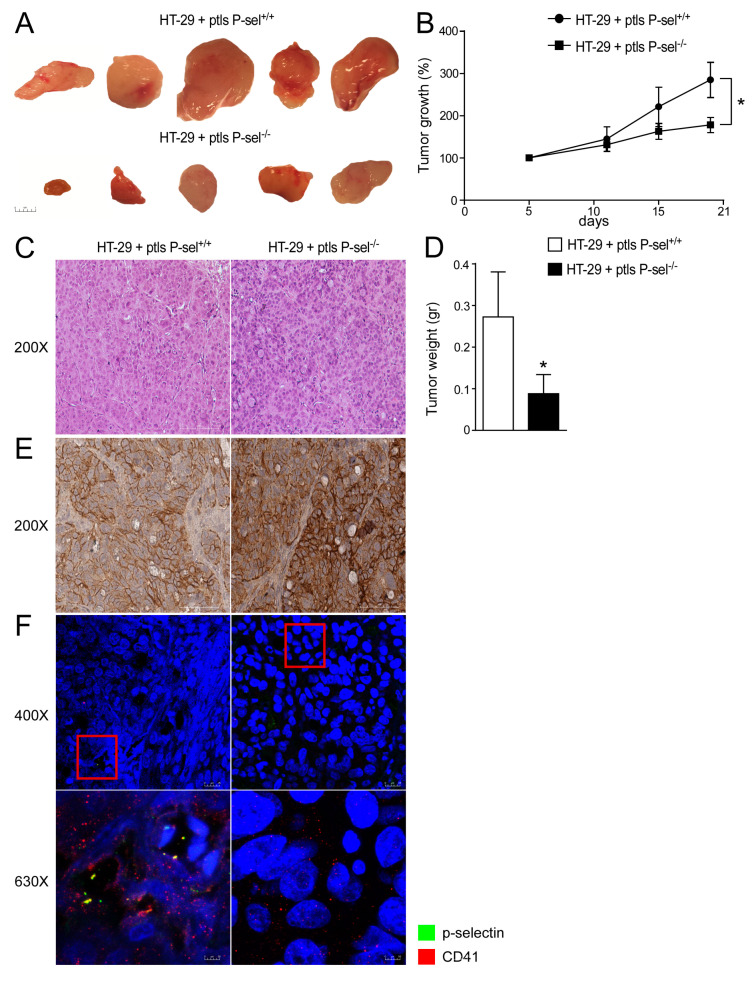
P-selectin KO platelets injection reduced tumor growth in xenograft tumor model. (**A**) Athymic nu/nu mice were injected subcutaneously with HT29 cells and platelets isolated from P-sel^−/−^ and P-sel^+/+^ mice were administered directly into the tumor mass every 7 days. Gross morphology of HT-29 cells treated with P-sel^+/+^ platelets and HT-29 cells treated with P-sel^−/−^. (**B**) Tumor growth (%) curves showed a reduction in the expansion of tumors injected with P-sel^−/−^ platelets (*n* = 10 mice per group). (**C**) Histology was assessed by H&E staining and was observed by light microscopy (magnification, 200×). Representative specimens are shown. (**D**) Tumor weight (gr) was reported. The results are expressed as mean ± SEM, *n* = 20 tumors per group; * *p* ≤ 0.05. (**E**) Paraffin-embedded tumor specimens from HT-29 cells treated with P-sel^+/+^ platelets and HT-29 cells treated with P-sel^−/−^ platelets were immunoassayed with CD44 antibody (200× magnification). Representative specimens are shown. (**F**) The protein expression of P-selectin and CD41 was investigated by immunofluorescence and confocal microscopy analysis in paraffin-embedded sections from tumors injected with P-sel^−/−^ or control platelets. P-selectin in green and CD41 in red. Representative images are shown. Magnification 630×.

In order to evaluate the tumor-promoting role exerted by platelets in CRC cells and the role of P-selectin in platelets-cancer cells crosstalk, we used a xenograft tumor model. Specifically, an HT-29 cell suspension was subcutaneously injected into the subscapular region of an athymic (nu/nu) mouse. Subsequently, a wild-type or P-selectin KO platelets suspension was directly injected into the tumor-bearing mice in each group every 7 days for a total of three injections per mouse over a time frame of 21 days. The tumor growth curves revealed a significant delay in the expansion of tumors treated with platelets isolated from the P-selectin KO mice (Figure 2A,B). In addition, as shown in Figure 2A,D, the injection of P-selectin KO platelets significantly reduced the tumor weight compared to the control group.

Furthermore, we evaluated the expression of CD44 (P-selectin receptor) in HT-29 cells and the presence of platelets in xenograft tumors. HT-29 cells treated with P-selectin KO or control platelets exhibited CD44 protein expression (Figure 2E). We observed that in tumors treated with platelets isolated from the control mice, there were several platelets linked to HT-29 cells compared to tumors treated with P-selectin KO platelets, where the absence of P-selectin marked platelets are observed, as expected (Figure 2F). These data support the role of P-selectin in the direct platelets-cancer cells crosstalk and the relevance of the interaction between platelets and colon cancer cells in intestinal tumorigenesis.

**Figure 3:**
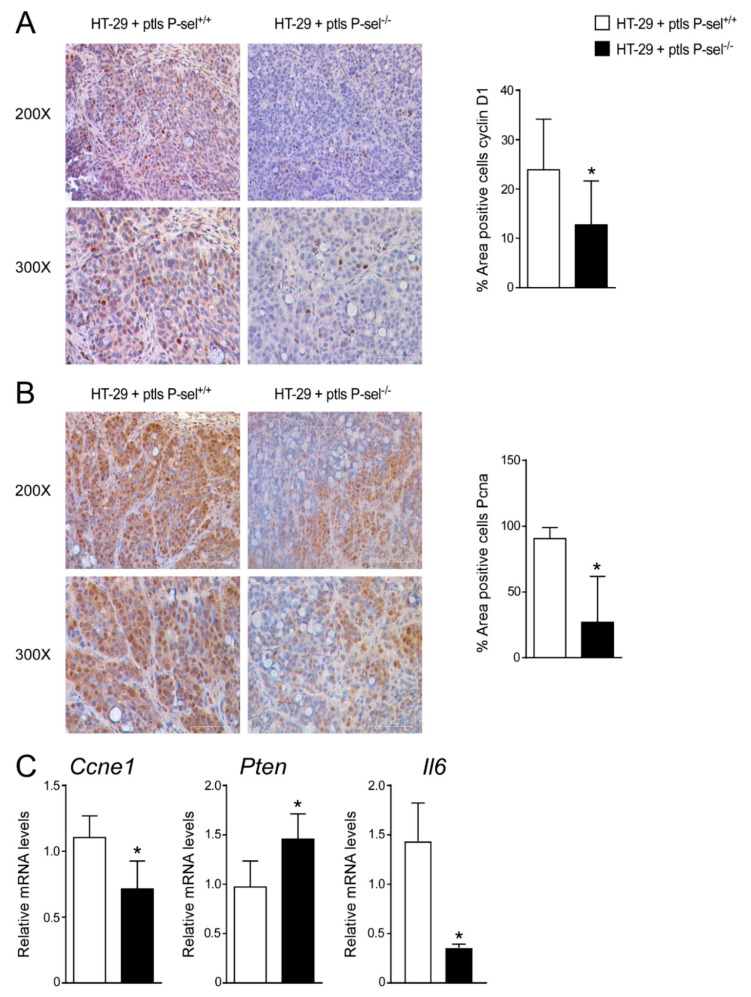
Treatment with P-selectin KO platelets reduced cell cycle progression in xenograft tumor model. Paraffin-embedded tumor specimens from HT-29 cells treated with P-sel^+/+^ platelets and HT-29 cells treated with P-sel^−/−^ platelets were immunoassayed with (**A**) cyclin D1 antibody (200× and 300× magnification) and (**B**) anti-Pcna antibody (200× and 300× magnification). Representative specimens are shown. Cyclin D1 and Pcna staining per field was quantified by ImageJ software and reported as percentage per field. Comparison of HT-29 cells treated with P-sel^+/+^ platelets and HT-29 cells treated with P-sel^−/−^ platelets (*n* = 8/group) was performed using the Student’s t-test. Results are expressed as mean ± SEM (* *p* < 0.05). (**C**) Gene expression analysis of Ccne1, Pten and Il6 in HT-29 cells treated with P-sel^+/+^ platelets and HT-29 cells treated with P-sel^−/−^ platelets (*n*= 20 tumors per group). Cyclophilin was used as a housekeeping gene to normalize data. The results are expressed as mean ± SEM. Statistical significance (* *p* < 0.05) was assessed by the Student’s *t*-test.

To explore the mechanisms underlying platelets mediated colon cancer cells’ proliferation, we examined if any alteration of cell cycle occurred, by analyzing the regulators of cell cycle progression (CyclinD1, CyclinE1 (Ccne1) and proliferating cell nuclear antigen (Pcna) and the tumor suppressor Pten (phosphatase and tensin homolog deleted on chromosome ten), which is frequently modified in human cancers, including breast, lung, prostate and bladder cancer [18]. In a xenograft tumor model, we observed a trend of reduction of the CyclinD1 and Pcna gene expression levels in HT-29 cells treated with P-selectin KO platelets compared to HT-29 cells treated with control platelets (Supplementary Figure S2). In this model, treatment with P-selectin KO platelets significantly changed cyclinD1 and Pcna protein accumulations, as well as Ccne1 and Pten gene expression compared to treatment with control platelets (Figure 3A–C). Furthermore, we analyzed the expression of Il6, a marker of inflammation, finding a significant downregulation of Il-6 gene expression levels in tumors treated with platelets isolated from the P-selectin KO mice compared to tumors treated with platelets isolated from the control mice (Figure 3C).

### 2.3. Lack of P-selectin Protects from Intestinal Carcinogenesis in Genetic Mouse Model of Tumor

**Figure 4:**
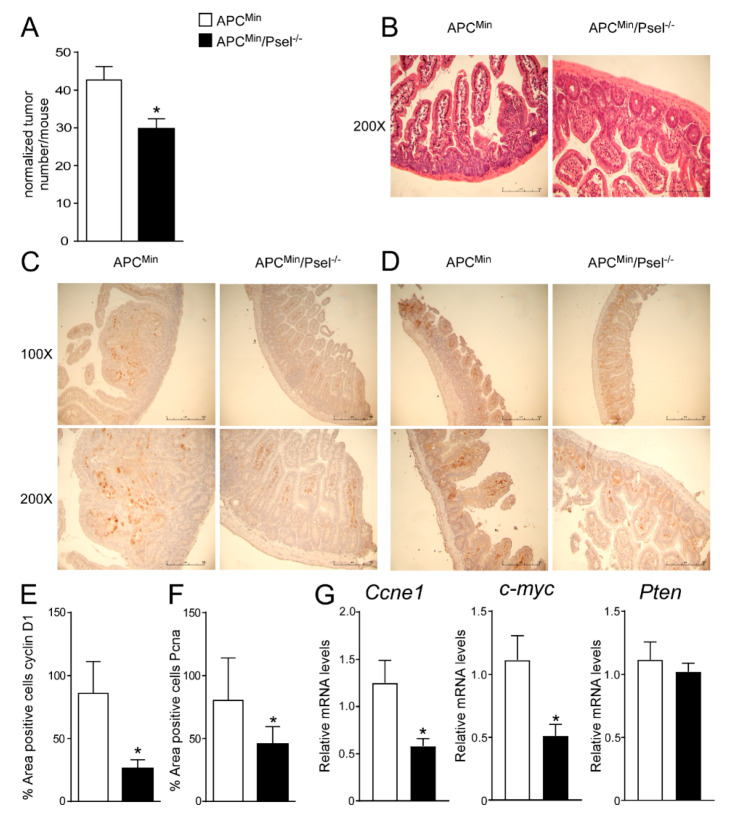
Role of P-selectin in genetic mouse model of colon cancer. (**A**) Total number of tumors was counted in APC^Min^/P-sel^−/−^ and APC^Min^ mice. Results are the average ± standard error of the mean (SEM), *n* = 10 mice per group; * *p* ≤ 0.05. (**B**) Histology was assessed by H&E staining and was observed by light microscopy (magnification, 200×). Representative specimens are shown. Paraffin-embedded tumor specimens from APC^Min^/P-sel^−/−^ and APC^Min^ mice were immunoassayed with (**C**) cyclin D1 antibody (100× and 200× magnification) and (**D**) anti-Pcna antibody (100× and 200× magnification). Representative specimens are shown. (**E**) Cyclin D1 and (**F**) Pcna staining per field was quantified by ImageJ software and reported as percentage per field. To perform protein quantification 10 representative images were taken with a 200× magnification for each sample. The percentage of stained area/total area was measured. Values from all consecutive images for each sample were averaged. Comparison of tumor specimens from APC^Min^/P-sel^−/−^ and APC^Min^ mice was performed using T student’s test (*n* = 5 mice per group). Results are expressed as mean ± SEM (* *p* < 0.05). (**G**) Gene expression analysis of Ccne1, c-myc and Pten in APC^Min^/P-sel^-/-^ and APC^Min^ mice. Cyclophilin was used as a housekeeping gene to normalize data. The results are expressed as mean ± SEM. Statistical significance (* *p* < 0.05) was assessed by the Student’s *t*-test (*n* = 10 mice per group).

To address the tumor-promoting role of P-selectin in colon cancer, we used a genetic model of intestinal tumor formation. We crossed APC^Min/+^ with P-sel^−/−^ or control mice to obtain a new genetic mouse model in which the lack of P-selectin occurred in mice that spontaneously developed tumors. The APC^Min^/P-sel^−/−^ mice exhibited a significant decrease in tumor number compared to the APC^Min/+^ mice (Figure 4A). The histological examination (H&E) of the APC^Min^/P-sel^−/−^ mice intestine displayed a more preserved parenchyma than the APC^Min/+^ mice, whereas a disrupted intestinal structure was detected in the APC^Min/+^ mice (Figure 4B). Furthermore, in the APC^Min^/P-sel^−/−^ mice we observed a significant reduction in cyclinD1 and Pcna protein levels compared to the APC^Min/+^ mice (Figure 4C–F). In the APC^Min^/P-sel^−/−^ mice, we did not find a significant difference in *Pten* and *Il6* gene expression levels and we detected a reduction in *c-myc* and *Ccne1* gene expression levels compared to the APC^Min/+^ mice (Supplementary Figure S3, Figure 4G), highlighting that the absence of P-selectin reduces tumor growth after pro-carcinogenic events. All together, these data point to the importance of platelets in tumor induction and progression in genetic, non-inflammatory models.

### 2.4. P-selectin KO Mice Are Protected from Chronic Colitis-Associated Colorectal Carcinogenesis

**Figure 5:**
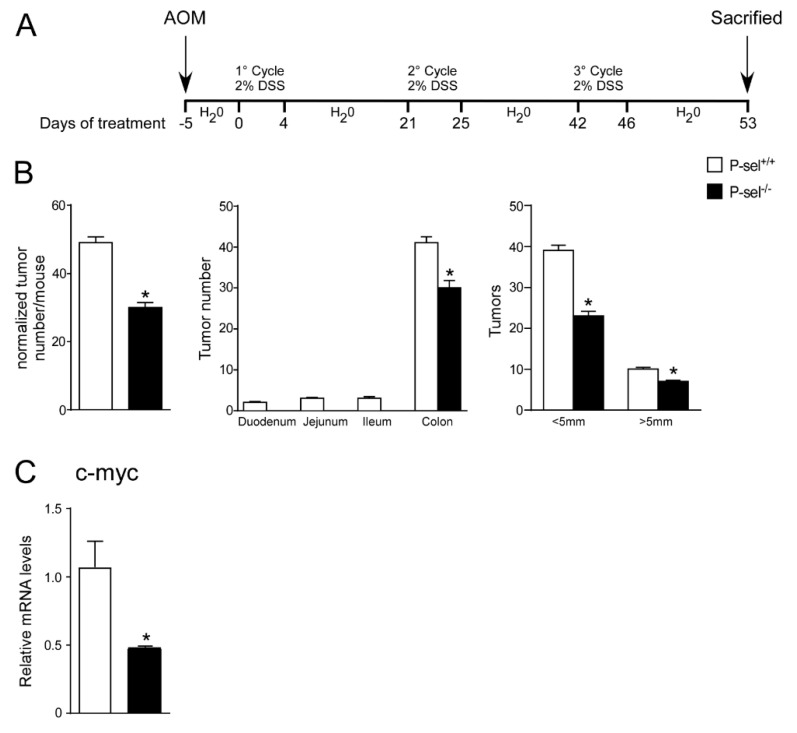
P-selectin KO mice are protected from chronic colitis-associated colorectal carcinogenesis. (**A**) P-sel^-/-^ (*n* = 10) and P-sel^+/+^ (wild-type; *n* = 10) mice were treated for a colitis-associated colorectal carcinogenesis model through one intraperitoneal azoxymethane (AOM) injection and 3 cycles of 2% dextran sulfate sodium (DSS) in drinking water. (**B**) Total number of tumors was counted. The diameter of each tumor was measured. The number of tumors with a diameter <5 mm and >5 mm was significantly reduced in P-sel^-/-^ mice showing that these mice are protected from colon cancer formation. Results are the average ± standard error of the mean (SEM); * *p* ≤ 0.05 (**C**) Gene expression analysis of c-myc in P-sel^-/-^ and P-sel^+/+^ mice. Cyclophilin was used as a housekeeping gene to normalize data. The results are expressed as mean ± SEM, * *p* ≤ 0.05.

In order to address the tumor-promoting role of P-selectin also in inflammatory models of colon cancer, we subjected P-selectin KO and the control wild-type (C57J/Bl6) mice to a model of chemically-induced colitis carcinogenesis [19]. To induce tumor formation, an intraperitoneal azoxymethane (AOM) injection was followed by three cycles of dextran sulfate sodium (DSS) treatment, as shown in Figure 5A. The P-selectin KO mice displayed a significant reduction in total tumor number and size with respect to the wild-type group (Figure 5B). The number of big (>5 mm) and small (<5 mm) tumors was significantly reduced in the P-selectin KO mice compared to the control mice (Figure 5B). C-myc overexpression is a fundamental oncogenic mechanism in several cancers, including colon cancer [20,21]. In the P-selectin KO mice we did not find significant difference in *Cyclin D1*, *Ccne1*, *Pten, Pcna* and *Il6* gene expression levels (Supplementary Figure S4) and we showed a reduction of c-myc gene expression compared to the control mice (Figure 5C). These data confirm the relevance of platelets in the tumor microenvironment and the prominent role of P-selectin to direct platelets-cancer cells crosstalk also in inflammation-induced colorectal tumorigenesis.

## 3. Discussion

The present study evaluated the relevance of platelets in the microenvironment of colorectal tumors and the prominent role of P-selectin to direct platelets-cancer cells crosstalk. First, we showed in the xenograft tumor model of colon cancer that at the variance of control platelets when P-selectin ablated platelets were inoculated in HT-29 tumors, they were not able to induce tumor growth. Furthermore, we demonstrated that the lack of P-selectin decreased tumor growth in genetic mouse models and in chemically-induced colitis colorectal carcinogenesis. 

The tumor microenvironment plays a pivotal role to modulate the ability of cancer cells to proliferate, to access the vasculature and to metastasize [22]. It has been shown that pharmacological or genetic depletion of blood platelets reduced tumor metastasis formation [23,24]. Interestingly, in a cardiovascular prevention trial, Rothwell et al. observed that patients taking aspirin at doses sufficient to block platelets function showed a reduced risk for cancer metastasis [25]. Several studies demonstrated that platelets promote metastasis via different mechanisms, such as the generation of platelets aggregates, the adhesion of cancer cells to the endothelium, the promotion of tumor vascularization and the tumor cell migration into the bloodstream [26,27]. Guillem-Llobat et al. observed that human platelets co-cultured with human colon carcinoma cells HT-29 promoted EMT contributing to the metastasis formation [28]. Furthermore, in HT-29 cells, platelets-cancer cells’ direct interaction induced COX-2-dependent PGE2 production leading to the regulation of p21 and cyclin B1 gene expression and the EMT promotion [27]. In the lungs, it has been shown that the contact between cancer cells and platelets is fundamental to induce pro-metastatic gene expression, EMT-like transformation and extravasation [10]. The open questions are the type of platelets-cancer cells’ interaction in colon cancer induction and/or development. 

Recently, in colitis-associated cancer, it has been observed that platelets represent a source of serum amyloid A that promotes myeloid cell-dependent immunosuppression leading to cancer development [29]. In the present work, we focused on CRC, a solid tumor closely related to platelets activities. Clinical studies and meta-analysis of randomized clinical trials have demonstrated that the administration of low-doses of aspirin, able to inhibit platelets function, decreased the risk of developing esophagus, stomach and colon cancers [25,30,31]. In chronic colitis-associated colorectal carcinogenesis, as well as in xenograft adenocarcinoma and in the genetic mouse model of CRC, we observed the crosstalk between platelets and colon cancer cells and we underscored the pivotal role of P-selectin in orchestrating this contact. P-selectin has been suggested as an important molecule for the adhesion of cancer cells [32]. At the same time, CD24, the P-selectin ligand, has been found in several human carcinomas [33,34]. Furthermore, CD44, another P-selectin ligand, has been shown in several colon cancer studies [14,28,35]. Guillem-Llobat et al. observed that coculturing HT-29 cells with human platelets promoted the mesenchymal-like cancer cells transition, cell mobility and proaggregatory action on platelets [28]. Alves et al. demonstrated the role of CD44 as a functional P-selectin ligand in colon adenocarcinoma cells [14]. Moreover, Hanley et al., through microbeads coated with CD44 immunoprecipitated from carcinomas, observed that variant isoforms of CD44 retained P-selectin binding activity [35]. In in vitro models of adenocarcinoma, P-selectin binding to cancer cells promoted their adhesion to fibronectin through the activation of p38MAPK and PI3-K signaling [36]. Moreover, Korniluk et al. observed a correlation between soluble P-selectin levels and the progression of CRC and the highest concentration of soluble P-selectin in patients with CRC and liver metastasis [37]. P-selectin KO mice injected with LS180 colon cells showed a reduction in tumor growth rate and in lung metastasis formation, suggesting that the P-selectin KO mice platelets did not adhere to tumor-cell surface receptors [16]. In a model of lung colony formation, it has been observed that thromboxane A2 receptor signaling enhanced tumor colonization via P-selectin-mediated platelets-cancer cells crosstalk [38]. Moreover, in insulinoma and CRC mouse models, P-selectin deletion and soluble P-selectin blocked platelets deposition within tumors and the secretion of VEGF, leading to the reduction of angiogenesis and tumor growth [39,40]. Recently, Wang et al. demonstrated that platelets expressing P-selectin enhance the ability of bone-marrow mesenchymal stem cells (BM-MSCs) to promote cancer metastasis. The supernatants of the BM-MSCs, when stimulated by platelets, enhance the expression of c-Myc in gastric tumor cells [41]. Furthermore, Mitrugno A. and colleagues demonstrated that in cancer cell lines (SW480 and PANC-1) proliferation was enhanced through human platelets via the upregulation and activation of c-myc. The ability of platelets to promote c-myc expression and cancer cell proliferation was reverted by aspirin [42]. Based on these recent data, it is conceivable that platelets-cancer cell interaction via P-selectin induces c-myc expression in cancer cells to promote cancer proliferation. Hence, the absence of P-selectin or the use of aspirin that reduces platelets activation and function inhibits platelet cancer cells crosstalk reducing tumor development.

Potential limitations of our study need to be appreciated. We conducted the experiment with human samples with a small number of participants. A xenograft mouse model was performed with platelets isolated from the P-selectin KO mice and wild-type using a single colon cancer HT-29 cell line.

In the present study, we found that P-selectin deficiency inhibited the direct interaction between platelets and colon cancer cells, thus reducing tumor growth by modulating the expression of genes involved in cell cycle progression, such as CyclinD1, Ccne1 and Pcna. Interestingly, both in the genetic mouse model (APC^Min^/P-sel^−/−^ mice) and in the inflammation-dependent cancer development model (AOM/DSS), we observed that the deletion of P-selectin inhibited tumor development mediating platelet adhesion to tumor cells. Furthermore, using P-selectin KO mice models and injecting platelet suspension directly into the tumor-bearing mice, we confirmed that platelets-cancer cells’ interaction has a pivotal role in cancer promotion and that platelets’ adhesion to cancer cells via P-selectin is necessary for the observed phenotype.

## 4. Material and Methods

### 4.1. Mice

Adenomatous polyposis coli APC^Min/+^/P-sel^−/−^ mice were generated by crossing pure strain APC^Min/+^ mice obtained from The Jackson Laboratory (Bar Harbor, ME, USA) with pure strain C57B6J/P-selectin^−/−^ mice kindly provided by Dr V. Evangelista (Chieti, Italy). All mice were housed under pathogen-free conditions in a temperature-controlled room (23 °C) on a 12 h light/dark cycle and fed a standard rodent chow diet and water ad libitum. 

### 4.2. Patients

We obtained tissue (tumors and normal intestinal mucosa) samples from the Pathology Section of Department of Emergency and Organ Transplantation (University of Bari, Bari, Italy). Six patients with colon adenocarcinoma and 4 control subjects were recruited for a previous study after approval by the Ethical Committee of the Azienda Ospedaliero-Universitaria Policlinico of Bari, Italy. The baseline characteristics of subjects are described in Table 1. All subjects gave their written informed consent for the use of clinical data and samples for scientific research purposes.

### 4.3. Platelets Isolation

The platelets were isolated from C57B6J or P-selectin^−/−^ mice whole blood and anti-coagulated with sodium citrate 3.8%. Platelet-rich plasma (PRP) was prepared by centrifugation of citrated blood at 200 g for 20 min. Platelets were washed with prostaglandin E1 (PGE1) (Sigma, Italy) and resuspended in an ice-cold, HEPES-Tyrode buffer (pH 7.4) containing 129 mM NaCl, 8.9 mM NaHCO_3_, 2.8 mM KCl, 0.8 mM KH^2^PO^4^, 56 mM dextrose, 10 mM HEPES. Immediately before platelet injection, 1 mmol/L MgCl^2^, 1 mmol/L CaCl^2^ and RGD peptide (GRGDNP) (Sigma, Italy) were added to the cells [43].

### 4.4. Xenograft Mouse Model

A colorectal cell suspension (100 µL; 1 × 10^7^ HT29 cells from American Type Culture Collection, Manassas, VA, USA) was injected subcutaneously into the subscapular region of an athymic CD1 mice (The Jackson Laboratory, Bar Harbor, ME, USA) as previously described [44]. The animals were treated with platelets isolated from P-selectin^−/−^ or wild- type mice directly injected into the tumor. For xenograft treated with mouse platelets, mice were divided randomly into 2 groups, each group containing 10 mice (i.e., 20 tumors) as follows: P-selectin^−/−^ platelets group and P-selectin^+/+^ platelets group (control group). Subsequently, 100 µL of platelets suspension (10^6^ platelets) was injected with a 27-gauge needle directly into the tumor-bearing mice in each group every 7 days for a total of 3 injections per mouse. Tumor growth was measured as follows: long diameter (a) and short diameter (b) were measured using a digital vernier caliper, and volumes (V) of the tumors were calculated according to the formula V = ½·a·b^2^, and a tumor growth curve was drawn. Tumor weight (gr) was measured using a calibrated analytical balance. All the mice then were sacrificed and tumors were harvested for further analysis. 

### 4.5. Colitis Carcinogenesis Model

For the chemically-induced colitis carcinogenesis model [19], 20 pathogen-free, 16-week-old male C57B6J (*n* = 10) and P-selectin^−/−^ (*n* = 10) mice were injected intraperitoneally with 12 mg/kg body weight of AOM dissolved in 0.9% NaCl. Five days later, 3% DSS was given in the drinking water over 5 days, followed by 16 days of regular water. This cycle was repeated 3 times and body weight was measured at the end of each cycle.

### 4.6. RNA Extraction

Total RNA was isolated by a Qiazol reagent (Qiagen) following the manufacturer’s instructions. To avoid possible DNA contaminations, the RNA was treated with DNase I (Thermo Fisher Scientific). RNA purity was checked by spectrophotometer and RNA integrity by examination on agarose gel electrophoresis. cDNA was synthesized retrotranscribing 4 μg of total RNA in a total volume of 100 μL using a High Capacity DNA Archive Kit (Thermo Fisher Scientific) following the manufacturer’s instructions.

### 4.7. Real-Time Quantitative PCR

qPCR assays were conducted in triplicate wells for each sample. Baseline values of amplification plots were set automatically, and threshold values were kept constant to obtain normalized cycle times and linear regression data. The reaction mixture per well used were as follows: 10 AL Power Syber Green (Thermo Fisher Scientific), 2.4 AL of primers at the final concentration of 150 nmol/L, 4.6 AL RNAase free water, and 3 AL cDNA (60 ng). For all experiments, PCR conditions used were as follows: denaturation at 95 °C for 10 min, followed by 40 cycles at 95 °C for 15 s, then at 60 °C for 60 s. Quantitative normalization of cDNA in each sample was performed using cyclophilin as an internal control. Validated primer sequences for RTqPCR are: Cyclin E1 FW GACCCACAGAGACAGCTTGGA; RV GTTCAGACAAACATGGCTTTCTTTG; c-myc FW TGTATGTGGAGCGGTTTCTCA; RV CTGGTAGGAGGCCAGCTTCT; Il6 FW ATGCTTCCAATCTGGATTCAATG; RV CTCAAACTCCAAAAGACCAGTGATG; cyclophilin A FW CCTTCACTTTGCCAAACACCAC; RV CATCTGCACTGCCAAGACTGAG; cyclophilin B FW GGCCAACGATAAGAAGAAGGG; RV ACAAAATTATCCACTGTTTTTGGAACA. For Pten RTqPCR we used an IDT validated primer (code Hs.PT.53.26587300). PCR assays were performed in 96-well optical reaction plates using the QuantStudio5 machine (Thermo Fisher Scientific). All reactions were run in triplicate. Relative quantification was conducted using the ΔΔCT method.

### 4.8. Histology and Immunohistochemistry

Tissue specimens were fixed in 10% formalin for 12 to 24 h, dehydrated, and paraffin embedded. Four µm thick sections were stained with hematoxylin-eosin (H&E) following standard protocols. The sections were subjected to antigen retrieval by boiling the slides in sodium citrate pH 6 (Sigma Aldrich, Milan, Italy) for 15 min. The sections were permeabilized in a phosphate-buffered saline (PBS) with 0.25% TritonX-100 for 5 min and were sequentially incubated for 10 min at room temperature in a protein blocking solution (Dako, Glostrup, Denmark) and overnight at 4 °C with the primary antibodies (anti-Pcna, sc-7907, Santa Cruz Biotechnology, Santa Cruz, CA, USA; or anti-cyclin D1, ab16663, Abcam, Cambridge, UK); or anti-CD44, ab6124, Abcam, Cambridge, UK). The sections were washed for 15 min in PBS and incubated for 25 min at room temperature with a DAKO real EnVision detection system Peroxidase/DAB^+^ (Dako, Glostrup, Denmark) according to the manufacturer’s instructions. After washing in PBS, the peroxidase reaction was initiated by incubation with DAB (Dako, Glostrup, Denmark). Coverslips were mounted with Permount and evaluated under a light microscope. Image processing was performed using ImageJ software. For each sample, 10 representative images were taken with a 200× magnification. The percentage of stained area/total area was measured. Values from all consecutive images for each sample were averaged. For negative controls, a 1% nonimmune serum in PBS replaced the primary antibodies.

### 4.9. Tissue Immunofluorescence and Confocal Laser Scanning Microscopy

The protein expression of CD62-P (P-selectin), CD41 and CD44 was evaluated by indirect immunofluorescence and confocal microscopy analysis. Paraffin-embedded sections were permeabilized in a phosphate-buffered saline (PBS) with 0.05% TWEEN-20 for 5 min, washed in PBS and then blocked with a 2% goat serum in PBS for 1 h at room temperature (RT). The sections were incubated overnight (ON) in a humidified chamber at 4 °C with a primary antibody against CD62-P (1:500) (ab6632, Abcam, Cambridge, UK), CD41 (1:100) (ab63983, Abcam, Cambridge, UK) or CD44 (1:500) (ab6124, Abcam, Cambridge, UK) following incubation for 1 h with the appropriate secondary antibody (Alexa Fluor 488 goat anti-mouse, 1:200, A32723, Thermo Fisher Scientific; Alexa Fluor 555 goat anti-mouse, 1:200, A32727, Thermo Fisher Scientific). The sections were counterstained with To-Pro (Thermo Fisher Scientific), mounted in Fluoromount (Biomeda Corp, Foster City, CA, USA) and sealed with nail varnish. Negative controls were performed by omitting the primary antibodies. Specific fluorescence was acquired by a Leica TCS SP8 (Leica, Wetzlar, Germany) confocal laser-scanning microscope using a 630× objective lense and 5× optical zoom. 

### 4.10. Statistical Analysis

All results are expressed as mean ± SEM. Statistical analysis was executed using GraphPad Prism software (v5.0; GraphPad Software Inc., San Diego, CA, USA). Comparisons of the two groups were performed using the Student’s *t*-test. A *p* value of <0.05 was considered significant.

## 5. Conclusions

In conclusion, we show here the key role of platelets in intestinal tumor microenvironments. Platelets-cancer cells crosstalk via P-selectin is fundamental for tumor development. Indeed, platelets that do not adhere to cancer cells are not able to induce tumor growth. Moreover, in the absence of platelets-cancer cells’ interaction, there is a reduction both in genetic and inflammatory-based colon cancer initiation and promotion. Our results support the hypothesis that a strategy aimed to inhibit platelets’ adhesion to tumor cells are able to block tumor growth and could represent a novel therapeutic approach to colon cancer treatment.

## Figures and Tables

**Table 1:** Clinical characterization of the study population. Data are presented as mean ± SEM (standard error of the mean).

Clinical Variable	Control	Colon Adenocarcinoma
*n* (M:F)	4 (2:2)	6 (4:2)
Age (year)	54.8 ± 9.5	60.1 ± 10.1
Pathological staging	-	T3/T4
Lymph node status	-	N0/N2

## Data Availability

The data presented in this study are all contained within this publication.

## Supplementary Materials

The following are available online at https://www.mdpi.com/article/10.3390/cancers13164243/s1, Figure S1: Platelets/intestinal cells crosstalk in human intestine. The protein expression of p-selectin and CD44 was investigated by immunofluorescence and confocal microscopy analysis in paraffin-embedded sections from normal portions of colon and colon adenocarcinoma. p-selectin in green and CD44 in red. Representative images are shown. Magnification 400×, Figure S2: Gene expression analysis of Cyclin D1 and Pcna in HT-29 cells treated with P-sel^+/+^ platelets and HT-29 cells treated with P-sel^−/−^platelets. Cyclophilin was used as a housekeeping gene to normalize data. The results are expressed as mean ± SEM. Statistical significance (*p* < 0.05) was assessed by T student’s test (*n* = 20 tumors per group), Figure S3: Gene expression analysis of Il6 in APCMin/P-sel^−/−^ and APCMin mice. Cyclophilin was used as a housekeeping gene to normalize data. The results are expressed as mean ± SEM. Statistical significance (*p* < 0.05) was assessed by T student’s test (*n* = 10 mice per group), Figure S4: P-selectin KO mice are protected from chronic colitis-associated colorectal carcinogenesis. Gene expression analysis of Cyclin D1, Ccne1, Pten, Pcna and Il6 in P-sel^−/−^ and P-sel^+/+^ mice. Cyclophilin was used as a housekeeping gene to normalize data. The results are expressed as mean ± SEM (*n* = 10 mice per group).
