# Supplementary material for: Adhesion of Platelets to Colon Cancer Cells Is Necessary to Promote Tumor Development in Xenograft, Genetic and Inflammation Models
Source: Cancers (Basel). 2021 Aug 23;13(16):4243. doi: 10.3390/cancers13164243 (PMC8394609; doi:10.3390/cancers13164243)
Supplement: Supplementary file 1 [file cancers-13-04243-s001.zip › cancers-1309708-supplementary/Supplementary Figure legend.pdf]

S1. Supplementary figure legend

Supp. Fig. S1. Platelets/intestinal cells crosstalk in human intestine. The protein expression of p-selectin and CD44 was investigated by immunofluorescence and confocal microscopy analysis in paraffin-embedded sections from normal portions of colon and colon adenocarcinoma. p-selectin in green and CD44 in red. Representative images are shown. Magnification 400X.

Supp. Fig. S2. Gene expression analysis of Cyclin D1 and Pcna in HT-29 cells treated with P-sel+/+ platelets and HT-29 cells treated with P-sel-/-platelets. Cyclophilin was used as a housekeeping gene to normalize data. The results are expressed as mean ± SEM. Statistical significance ($P < 0.05$) was assessed by T student's test (N= 20 tumors per group).

Supp. Fig. S3. Gene expression analysis of Il6 in APCMin/P-sel-/- and APCMin mice. Cyclophilin was used as a housekeeping gene to normalize data. The results are expressed as mean ± SEM. Statistical significance ($P < 0.05$) was assessed by T student's test (N=10 mice per group).

Supp. Fig. S4. P-selectin KO mice are protected from chronic colitis-associated colorectal carcinogenesis. Gene expression analysis of Cyclin D1, Ccne1, Pten, Pcna and Il6 in P-sel-/- and P-sel+/+ mice. Cyclophilin was used as a housekeeping gene to normalize data. The results are expressed as mean ± SEM (N=10 mice per group).
