# Supplementary figures and images for: Adhesion of Platelets to Colon Cancer Cells Is Necessary to Promote Tumor Development in Xenograft, Genetic and Inflammation Models
Source: Cancers (Basel). 2021 Aug 23;13(16):4243. doi: 10.3390/cancers13164243 (PMC8394609; doi:10.3390/cancers13164243)

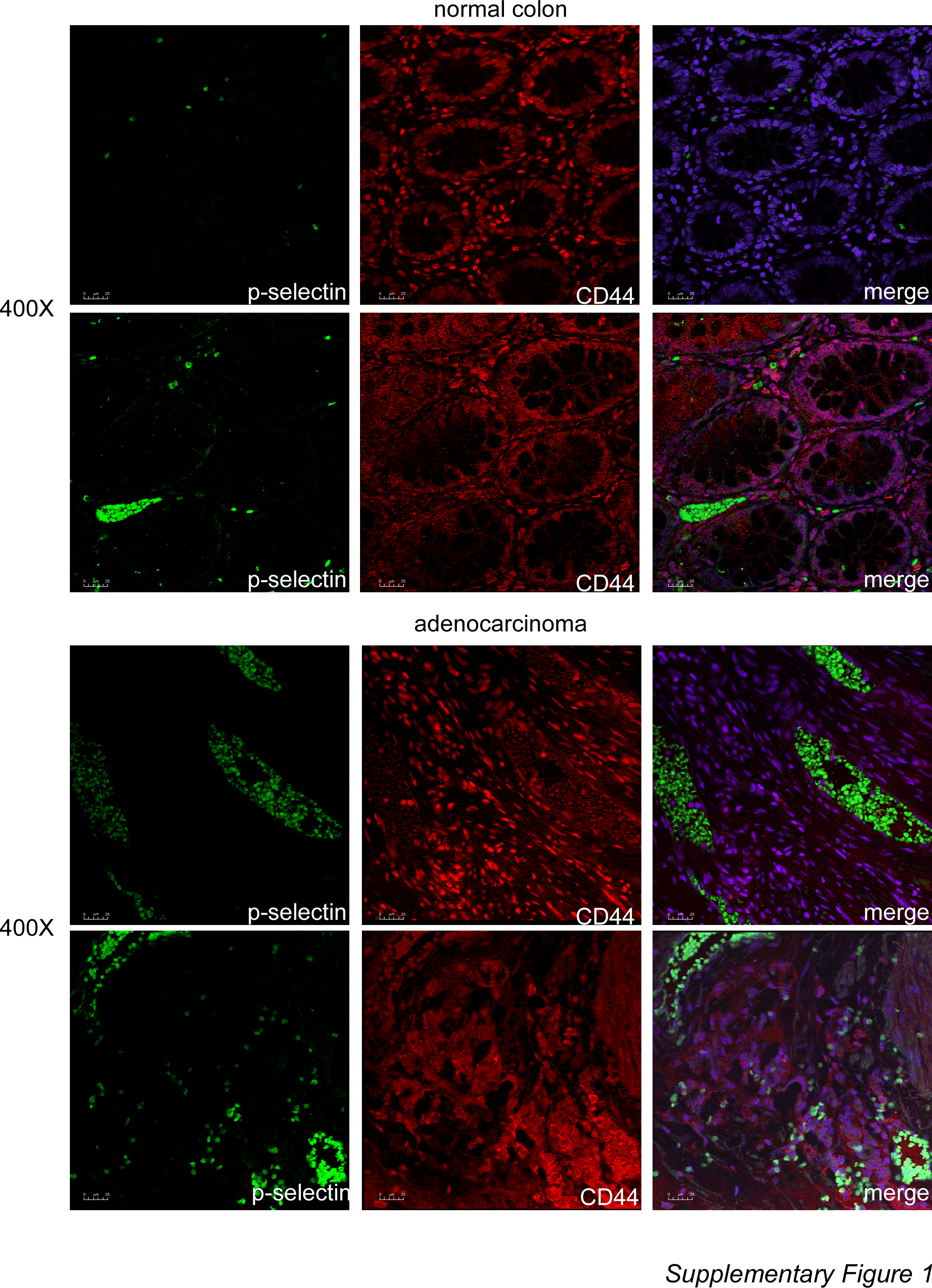

Supplement: Supplementary file 1 [file cancers-13-04243-s001.zip › cancers-1309708-supplementary/Supp Fig S1.tif]

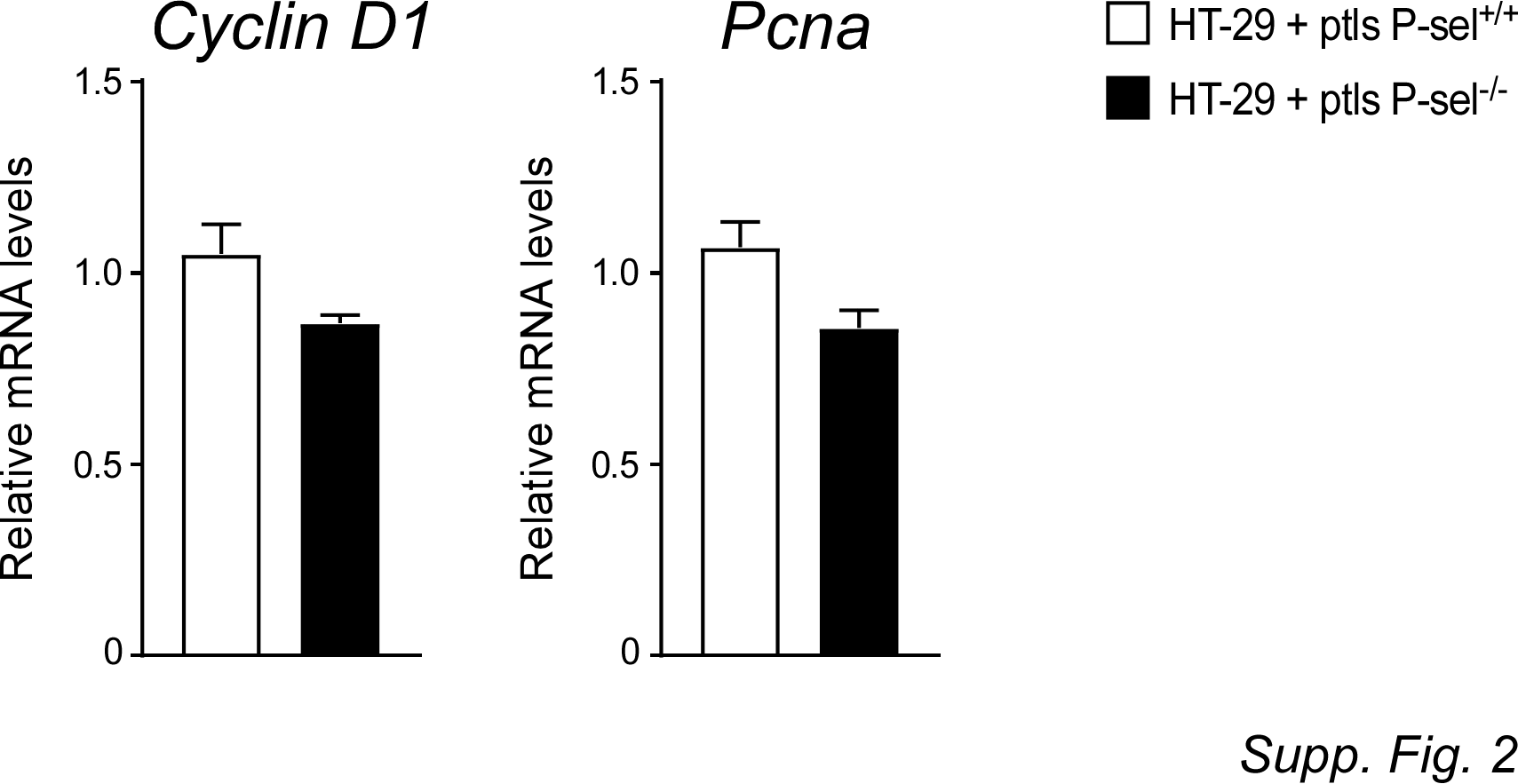

Supplement: Supplementary file 1 [file cancers-13-04243-s001.zip › cancers-1309708-supplementary/Supp Fig S2.tif]

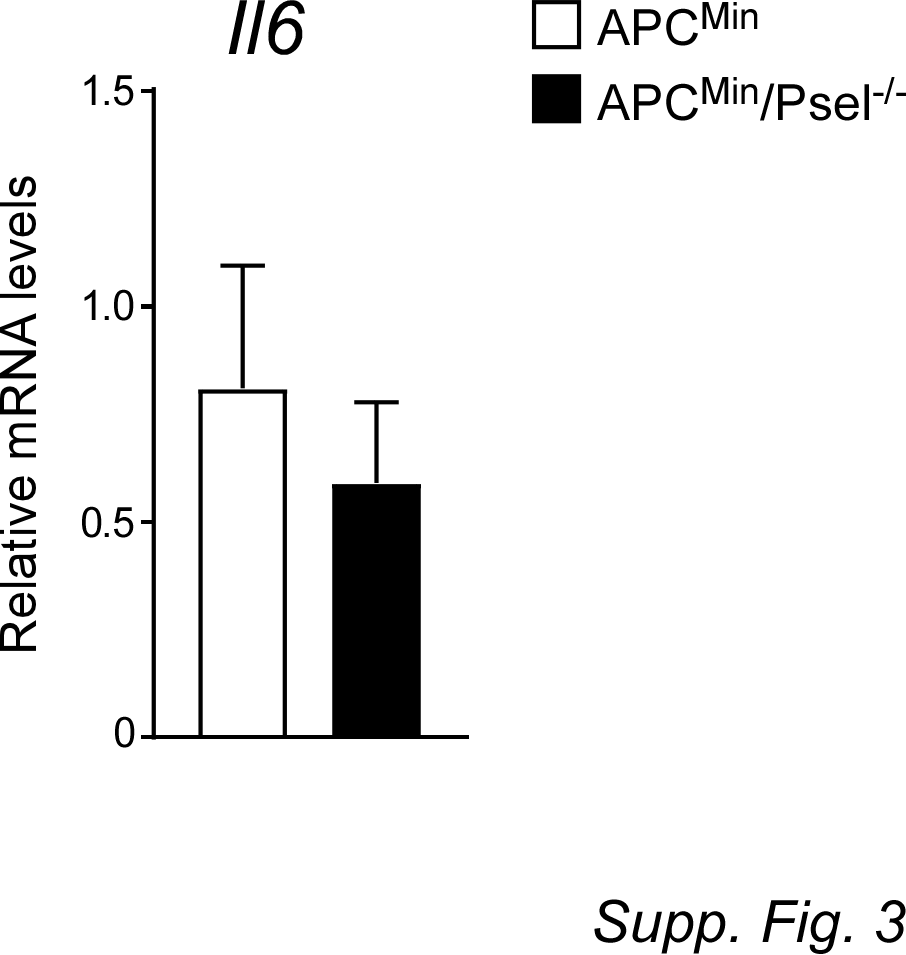

Supplement: Supplementary file 1 [file cancers-13-04243-s001.zip › cancers-1309708-supplementary/Supp fig S3.tif]

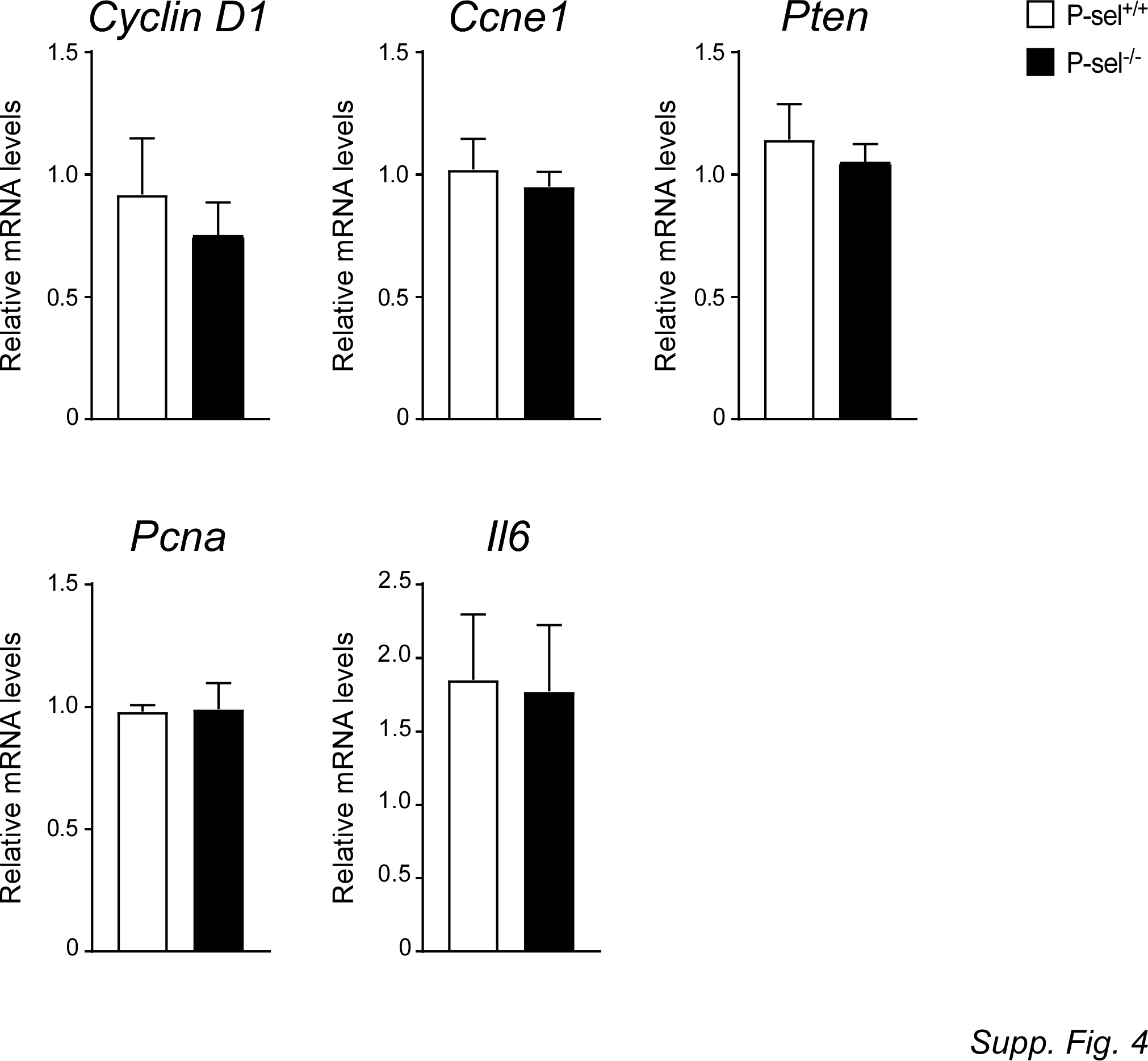

Supplement: Supplementary file 1 [file cancers-13-04243-s001.zip › cancers-1309708-supplementary/Supp Fig S4.tif]
